# Rolling bearing fault diagnosis method based on gramian angular difference field and dynamic self-calibrated convolution module

**DOI:** 10.1371/journal.pone.0314898

**Published:** 2024-12-31

**Authors:** Chunli Liu, Jiarui Bai, Linlin Xue, Zhengkun Xue

**Affiliations:** 1 School of Mechanical Engineering and Automation, University of Science and Technology Liaoning, Anshan, China; 2 School of Electronic and Information Engineering, University of Science and Technology Liaoning, Anshan, China; 3 School of Aerospace Engineering, Xiamen University, Xiamen, China; SRM Institute of Science and Technology (Deemed to be University), INDIA

## Abstract

To address the problem of insufficient feature extraction abilities of traditional fault diagnosis methods under conditions of sample scarcity and strong noise interference, a rolling bearing fault diagnosis method based on the Gramian Angular Difference Field (GADF) and Dynamic Self-Calibrated Convolution (DSC) is proposed. First, the GADF method converts one-dimensional signals into GADF images to capture nonlinear relationships and periodic information in time-series data. Second, a dynamic self-calibrated convolution module is introduced to enhance the feature extraction ability of the model. The DSC module dynamically adjusts the weights of parallel convolution kernels based on real-time data characteristics, effectively improving the feature extraction ability and generalization performance of the model. Finally, the proposed method is validated using bearing datasets from Huazhong University of Science and Technology and Harbin Institute of Technology, and is compared with other advanced models. The results show that the classification accuracy of the proposed method is basically above 90% when adding Gaussian white noise with a signal-to-noise ratio of -8 dB, which is a significant improvement of 6%-15% compared with other models. Therefore, the proposed method has excellent diagnostic performance in the rolling bearing fault diagnosis task under strong noise and small training samples.

## Introduction

The efficient and stable operation of mechanical equipment is crucial for enhancing production efficiency and ensuring safety. As a core component of mechanical equipment, the working conditions of rolling bearings directly affect the performance and reliability of the entire mechanical system [1, 2]. However, due to long-term work in the harsh conditions of high speeds and heavy loads, rolling bearings are prone to failures such as wear, cracks, and loosening. These faults not only reduce the performance and reliability of the mechanical system but also lead to equipment damage and production accidents, causing significant economic losses and safety risks [3].

Extensive research has been conducted to ensure the safe operation of mechanical systems and to promptly detect and diagnose bearing faults, leading to the proposal of various fault diagnosis methods. In the field of rolling bearing fault diagnosis, effectively extracting fault-related features from complex vibration signals is a key challenge. Traditional fault diagnosis methods include vibration analysis [4], sound analysis [5], and temperature monitoring [6]. While these methods can detect bearing faults to some extent, they are often dependent on specialized instruments and experienced technicians, with high data quality requirements, presenting limitations in practical applications.

In recent years, deep learning has achieved remarkable success in fields such as image recognition [7], speech recognition [8], and natural language processing [9] due to the rapid development of artificial intelligence technology. Inspired by these successes, researchers have increasingly attempted to apply deep learning techniques to rolling bearing fault diagnosis to improve diagnostic accuracy and efficiency [10, 11]. Compared with traditional methods, deep learning-based fault diagnosis methods offer greater intelligence and adaptability. Deep learning models can automatically learn and extract features from large amounts of data, effectively identifying and classifying complex fault types. Convolutional Neural Network (CNN) [12] is considered practical and effective in rolling bearing fault diagnosis, but still faces some challenges in practical applications. For instance, noise interference can cause significant changes in vibration signals, reducing the distinction of fault features and impairing the accuracy and reliability of fault diagnosis. To address this issue, researchers have begun exploring more advanced deep learning models.

Chen et al. [13] utilized convolution kernels of various sizes to capture multi-scale features of input data, aligning output features while ensuring shift invariance. Experimental results demonstrated excellent performance in classification accuracy and generalization ability. Wang et al. [14] innovatively designed a multi-scale denoising network by integrating dual-scale convolution blocks and wavelet-based denoising layers, effectively extracting multi-scale feature information from input data. Experiments demonstrated high diagnostic accuracy even in noisy environments. Ghorvei et al. [15] used graph convolutional neural networks for feature extraction of vibration signals under varying working conditions, which applied adversarial domain adaptation and local maximum mean discrepancy methods to reduce data distribution differences, achieving good classification results under varying conditions. Jia et al. [16] proposed a multi-scale residual attention CNN to suppress noise. This model uses multi-scale learning modules to obtain feature maps at different scales and efficiently assigns weights to all positions of different channels through residual attention modules to complete denoising operations, extracting more effective features in strong noise environments. Sun et al. [17] converted vibration signals into symmetrical snowflake images based on the symmetrized dot pattern, which effectively preserved the fault features and fully utilized the performance of CNN. Zhu et al. [18] optimized features extracted by multi-scale CNN using intra-class and inter-class constraints, improving sample distribution and finely adjusting through local sensitive discriminant analysis and particle swarm optimization strategy to enhance feature differentiation. Song et al. [19] used wide convolution kernels to quickly extract features and experimented with small convolution kernels to produce multi-layer nonlinear mappings, demonstrating excellent noise resistance and robustness. Liu et al. [20] proposed a denoising model based on vibration signal image features, converting one-dimensional vibration signals into asymmetric recurrence plots through wavelet decomposition, significantly improving noise resistance and diagnostic accuracy. Vashishtha et al. [21] conducted in-depth research on the hyperparameter selection of CNN, and innovatively proposed an amended gorilla troop optimization algorithm for optimally selecting the hyperparameters of the CNN. By converting one-dimensional signals into two-dimensional wavelet maps as CNN inputs, high-precision fault diagnosis was successfully realized. Shao et al. [22] used a multi-scale feature fusion CNN with a multi-stage attention scheme to extract low-noise features and minimize domain shift through correlated alignment distance, learning rich fault feature information with limited samples. Liu et al. [23] addressed the non-stationary nature of vibration signals by converting them into grayscale images and extracting features through multi-scale convolution and improved attention mechanisms, achieving high recognition accuracy with few training parameters. Ye et al. [24] improved the CNN model by introducing an attention mechanism and used variational mode extraction to eliminate noise interference, thereby enhancing fault recognition ability.

The above research indicates that deep learning technology holds significant potential in rolling bearing fault diagnosis, but it necessitates further investigation and enhancement to improve diagnostic accuracy and reliability. To address the issue of poor fault diagnosis performance under sample scarcity and strong noise interference, this paper proposes a rolling bearing fault diagnosis method based on GADF and DSC, termed the dynamic self-calibrated convolutional neural network (DSCNN). By converting vibration signals into GADF images, DSCNN effectively captures nonlinear relationships within time-series data, thereby better revealing fault characteristics. The dynamic self-calibrated convolution module is an innovative structure that dynamically adjusts convolution kernel weights according to input data characteristics, enhancing the adaptability of the model to bearing vibration signals under varying working conditions. Compared to traditional static convolution networks, DSCNN offers enhanced noise resistance and robustness, thereby effectively improving fault diagnosis accuracy and reliability.

## Theoretical basis

### Convolutional neural network

As a crucial branch of deep learning, CNN [25] has achieved remarkable achievements in recent years. Inspired by biological visual system research, particularly the working principles of the visual cortex, CNN exhibits exceptional performance in image processing, natural language processing, and speech recognition.

**Fig 1:**
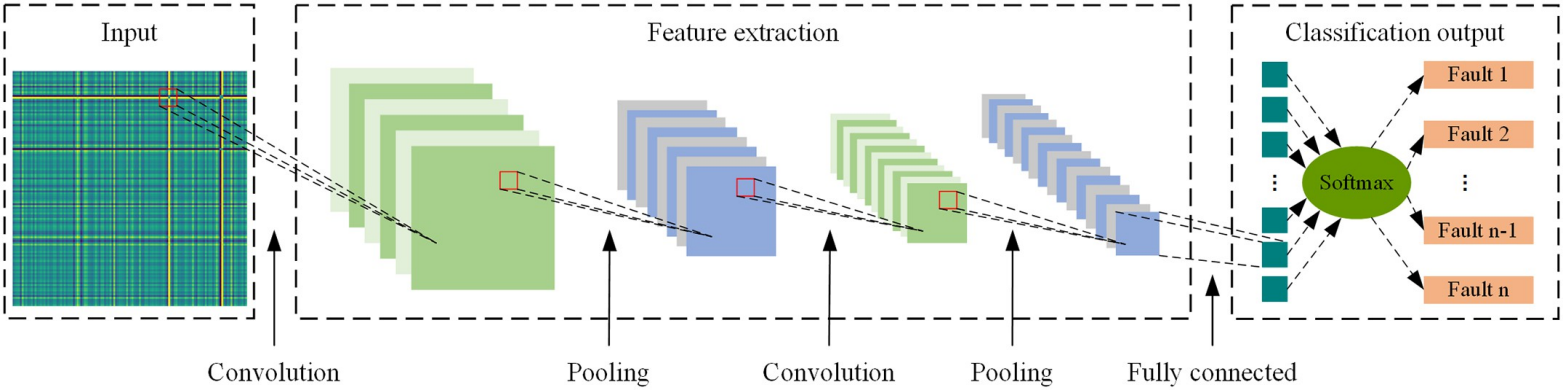
CNN structure.

The basic principle of CNN is to use convolutional operations to extract features from input data and progressively extract higher-level features through a network structure composed of stacked layers, ultimately achieving the understanding and classification of input data. This hierarchical feature extraction method makes CNN efficient and accurate in processing large-scale data. A typical CNN structure usually consists of multiple convolutional layers, pooling layers, and fully connected layers, as shown in Fig 1.

In the convolutional layers, the network extracts local features from input data through learnable convolution kernels. The pooling layers reduce the dimensionality of feature map output by convolutional layers, thereby reducing computational complexity and improving robustness. The fully connected layers integrate features extracted by convolutional and pooling layers for the final classification task. Through the layered structure, CNN can progressively learn more abstract and complex feature representations, enhancing model performance and generalization ability and effectively handling complex data.

The characteristics of CNN are mainly reflected in the following aspects. Firstly, local connections and weight sharing result in fewer parameters and improved generalization ability. Secondly, by stacking multiple convolutional and pooling layers, CNN can progressively extract feature representations of input data, achieving efficient representation of complex data. Additionally, CNN has certain robustness to translational variations of input data and can preserve positional information, which is crucial in many tasks. Finally, CNN structures can be flexibly adjusted based on specific tasks and datasets to meet the requirements of different application scenarios.

### Gramian angular field

In signal processing and data analysis, data visualization has always been a research focus. In recent years, Gramian Angular Field (GAF) [26] has provided an effective solution for converting one-dimensional signals into two-dimensional images as a data transformation method.

The core idea of GAF is to map one-dimensional time-series data to a polar coordinate system, calculate the angles or angular differences between adjacent data points, and store these values in a matrix. Finally, the matrix is converted into a two-dimensional Gramian Angular Summation Field (GASF) or Gramian Angular Difference Field (GADF) image. Compared to GASF, GADF focuses more on differences in time-series data, better capturing nonlinear relationships, making it more suitable for time-series data with nonlinear and non-stationary characteristics.

**Fig 2:**
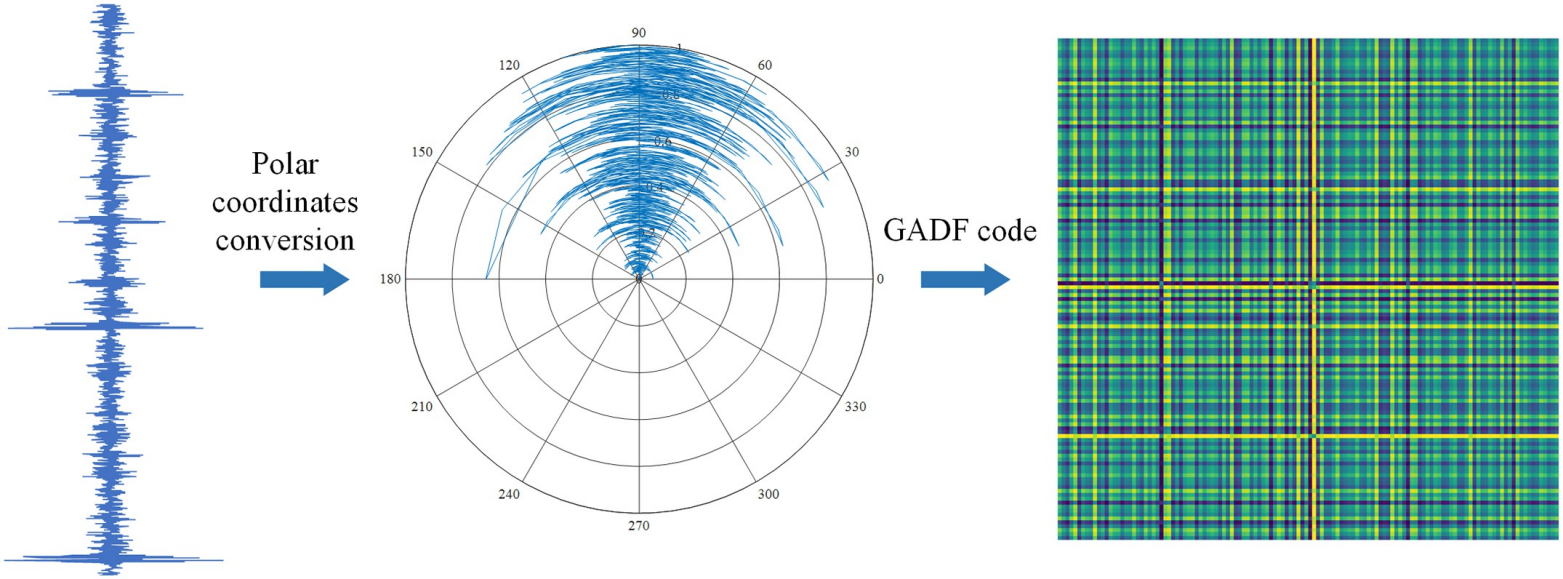
GADF conversion.

The GADF conversion process is shown in Fig 2. Assuming the given one-dimensional time-series data is *X* = {*x*_1_,*x*_2_,⋯,*x*_i_,⋯,*x*_*n*_}, the steps to convert it to a GADF image are as follows:

Step 1: To enhance the visual effects of the resulting GADF image, the data is normalized to the [-1, 1] range.
$${\tilde{x}}_{i}=\frac{\left(x_{i}-\text{max}\left(X\right)\right)+\left(x_{i}-\text{min}\left(X\right)\right)}{\text{max}\left(X\right)-\text{min}\left(X\right)}$$ (1)
Step 2: Convert the normalized data ${\tilde{x}}_{i}$ to polar coordinates, where each data point ${\tilde{x}}_{i}$ corresponds to a polar coordinate angle *θ*_*i*_, expressed as:
$$\left\{\begin{array}{ll} \theta_{i}= & \text{arccos}\left({\tilde{x}}_{i}\right)\\ r_{i}= & \frac{t_{i}}{N} \end{array}\right.$$ (2)
Where *r*_*i*_ is the distance from the positioning point to the origin of the polar coordinate system, *t*_*i*_ is the timestamp, and *N* is a constant factor. Therefore, for any data point, Eq (2) ensures a unique mapping result in the polar coordinate system.
  Step 3: Based on the polar coordinate system, construct a matrix *G* of *N × N*, where *G*(*i*, *j*) represents the sine value of the angle between data points *x*_*i*_ and *x*_*j*_, expressed as:
$$\begin{array}{l} G=\left[\begin{array}{ccc} \text{sin}\left(\theta_{1}-\theta_{1}\right) & \cdots & \text{sin}\left(\theta_{1}-\theta_{n}\right)\\ \vdots & \ddots & \vdots \\ \text{sin}\left(\theta_{n}-\theta_{1}\right) & \cdots & \text{sin}\left(\theta_{n}-\theta_{n}\right) \end{array}\right]\\ ={\left(\sqrt{I-X^{2}}\right)}^{\prime }\cdot X-X^{\prime }\cdot \sqrt{I-X^{2}} \end{array}$$ (3)
Step 4: Finally, the matrix *G* is converted into a GADF image, where the grayscale value of each pixel corresponds to the matrix value, achieving the conversion of one-dimensional time-series data to a two-dimensional image.

GADF images can intuitively reflect change patterns and periodic information in time-series data, which is significant for many data analysis tasks. In rolling bearing fault diagnosis, converting vibration signals into GADF images can better capture nonlinear relationships and periodic information, providing rich feature information for fault diagnosis models.

## Proposed method

### Self-calibrated convolution

**Fig 3:**
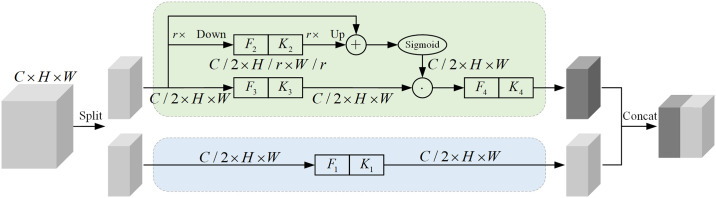
Self-calibrated convolution.

Self-calibrated convolution (SC) [27] is an innovative convolutional module for processing complex signals. Its structure is shown in Fig 3. Unlike traditional convolutions, SC breaks locality constraints by introducing heterogeneous convolutions and kernel communication, enables each spatial location to encode long-range contextual information, improving feature extraction and expanding the receptive field.

Traditional convolution operates in local regions, limiting the ability of the model to utilize long-range contextual information, affecting feature extraction quality. SC addresses this issue by incorporating heterogeneous convolutions. First, SC down samples the input to a low-dimensional embedding. Then, part of the convolution kernel transforms the low-dimensional embedding to calibrate the convolution transformations of other kernels. The communication between heterogeneous convolutions allows each spatial location to comprehensively perceive long-range contextual information, effectively expanding the receptive field of each spatial position.

### Cooperative compensation attention mechanism

**Fig 4:**
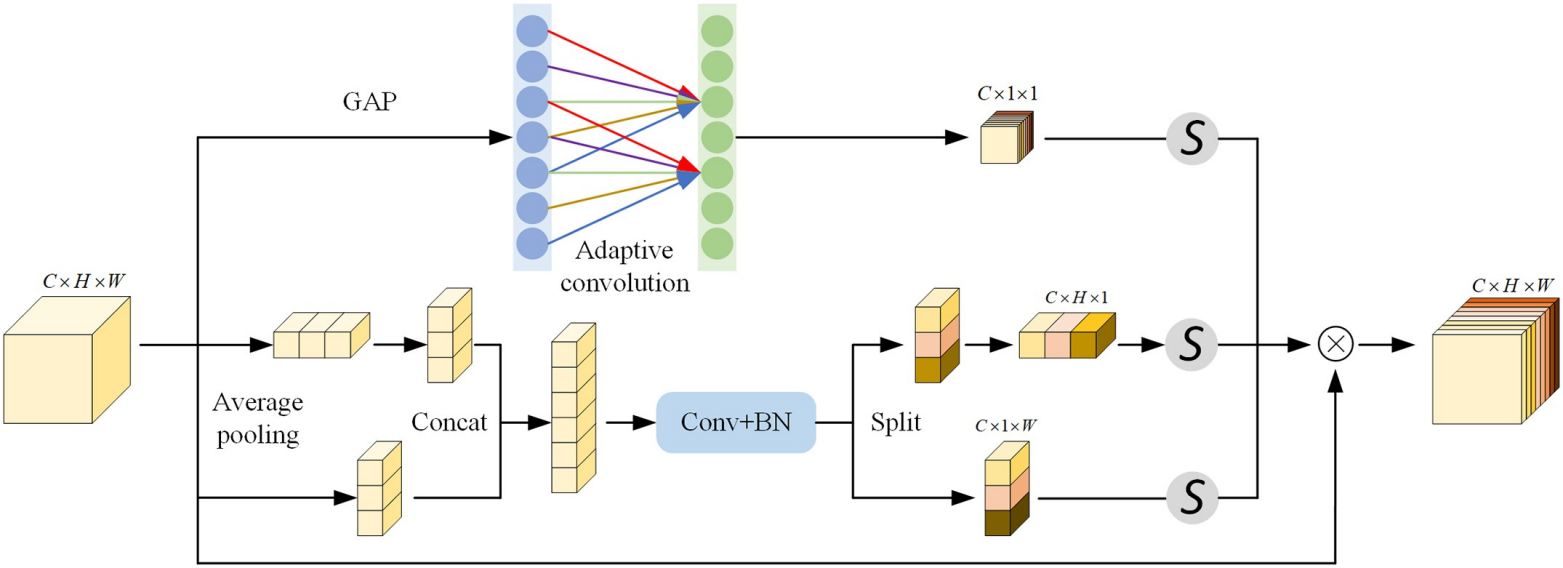
Cooperative compensation attention mechanism.

Traditional attention mechanisms typically focus on single-dimensional feature weights, which may not fully consider the comprehensive relationships between features, thereby limiting the performance of the model [28]. To address this issue, a cooperative compensation attention (CCA) mechanism is proposed. CCA simultaneously extracts channel and spatial weights, dynamically evaluating and assigning weights to different parallel SCC modules. Its structure is illustrated in Fig 4.

For each parallel SC module, the CCA deeply analyzes the complex interactions between features. CCA synchronously computes channel and spatial correlations, revealing the interrelationships between feature channels and accurately measuring the relationships between features at different spatial positions. The simultaneous computation ensures comprehensive analysis of feature relationships, crucial for capturing spatial structural information. The computed channel and spatial correlations are combined to calculate the final attention weights. These weights are used to weight the outputs of each SC module, synthesizing a more comprehensive and precise feature representation. This approach not only enhances the understanding of the features in the model, but also improves feature expression accuracy, thereby boosting overall model performance.

The advantage of CCA lies in its ability to consider correlations in both channel and spatial dimensions, providing a comprehensive assessment of feature importance. A comprehensive analysis enables deeper understanding of data features and dynamic adjustment of module weights based on data characteristics. With CCA, the model gains flexibility to adjust module weights in real-time according to the varying characteristics of the data, achieving feature complementarity and optimization, significantly enhancing the ability of the model to handle complex data distributions and diverse task requirements. Through dynamic adaptive adjustment, the model can more accurately capture key information in the data, exhibiting higher efficiency and accuracy in handling complex tasks.

### Model structure and parameters

Rolling bearing vibration signals often exhibit complex vibration characteristics, with dynamic changes in features over time and frequency. Traditional convolution operations cannot flexibly adjust convolution kernel parameters to adapt to the dynamic changes of the signals, and thus important detail information may be lost during the convolution process. To address this issue, a dynamic self-calibrated convolution (DSC) module is proposed to enhance the feature extraction and generalization abilities of the model.

The DSC module integrates convolution kernels of three different scales to optimize feature extraction efficiency. Each convolution kernel is assigned unique weights that are dynamically adjusted during training based on feature requirements. In DSC design, weight allocation is not static but determined by the importance of features extracted by each convolution kernel. Dynamic weight adjustment ensures the model optimizes the contribution of each convolution kernel according to feature effectiveness.

Specifically, the weight allocation strategy is highly flexible and responds in real-time to feature demands during training. Small-scale convolution kernels focus on extracting fine details, medium-scale kernels balance local features with broader information, and large-scale kernels capture comprehensive contextual information, crucial for enhancing noise resistance. Large convolution kernels integrate more local information, effectively smoothing noise interference in feature extraction and ensuring stable recognition of signal core patterns even in noisy environments.

DSC combines SC and CCA mechanisms, whose synergy provides extraordinary flexibility and precision in processing complex vibration signals. Through dynamic weight adjustment of SC modules by the CCA mechanism, DSC enhances adaptability and feature expression abilities, effectively handling nonlinear and non-stationary signals. The fine-tuning strategy significantly improves noise resistance, allowing DSC to effectively identify and extract key features in noisy environments, ensuring robustness and accuracy in practical applications. Additionally, the weighting strategy helps reduce overfitting risk, enhancing the generalization ability of the model, making it more adaptable to diverse working conditions and environmental changes.

**Fig 5:**
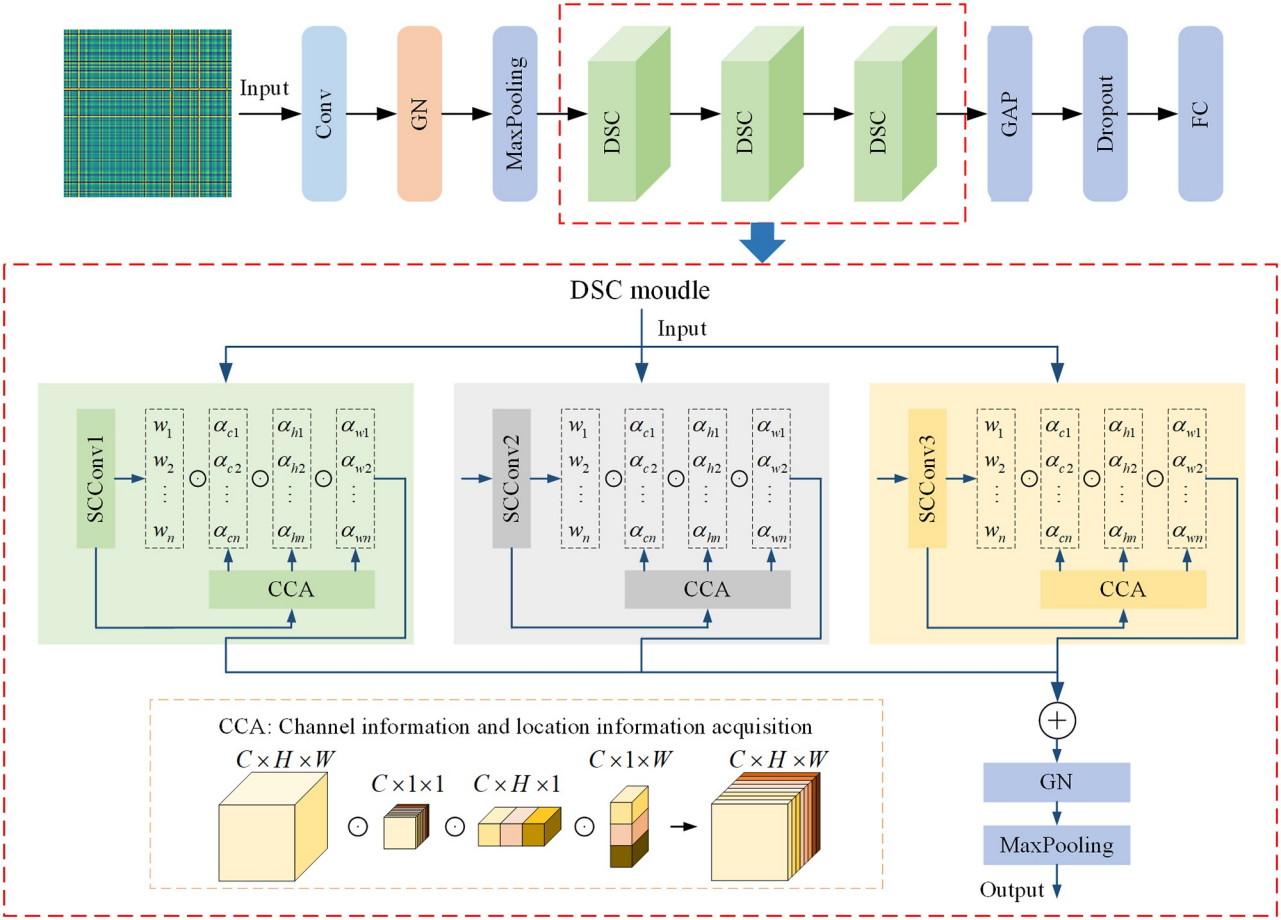
DSCNN model structure.

The proposed model structure is shown in Fig 5. First, the rolling bearing vibration signals are converted into GADF feature maps for processing. These feature maps are input into convolutional layers for feature extraction, followed by group normalization and max-pooling to enhance feature representation and stability, reducing feature map dimensions and computational load. The processed feature maps are then input into the DSC module for feature learning and fault diagnosis, and subsequently classified through GAP and fully connected layers. The hyperparameters of the DSCNN model are shown in Table 1.

**Table 1:** DSCNN model hyperparameters.

Layer Name	Hyperparameters
Input	Input size: [128, 128]
Convolution	Kernel size: [9, 9], Number: 64, Output: [128, 128]
Max Pooling	Kernel size: [2, 2], Output: [64, 64]
DSC1	Kernel size: [3, 3], [9, 9], [17, 17], Number: 64, Output: [64, 64]
DSC2	Kernel size: [3, 3], [9, 9], [17, 17], Number: 64, Output: [32, 32]
DSC3	Kernel size: [3, 3], [9, 9], [17, 17], Number: 64, Output: [16, 16]
GAP	—
Dropout	0.5
Fully Connected	—

During training, the parameters were optimized using Adam algorithm, with categorical cross-entropy as the loss function, ReLU as the activation function, an initial learning rate of 0.0001, and epoch set to 30. The implementation of the DSCNN is shown in Table 2.

**Table 2:** Implementation process of DSCNN.

Algorithm: Implementation process of DSCNN
Input: Raw vibration dataset *X* and corresponding labels *X*_*label*_. Output: Predicted bearing health states *Y*_*predict*_. 1. Data pre-processing: process the raw dataset to obtain the preprocessed data: *X*_*pre-processed*_ = *pre* − *process*(*X*). 2. Generate GADF Images: Convert the pre-processed data into Gramian Angular Difference Field (GADF) images: *X*_*GADF*_ = *Generate*-*GADF*(*X*_*pre-processed*_). 3. Dataset splitting: Split the GADF images and labels into training, validation, and test sets with a ratio 1:1:2: (*X*_*train*_, *X*_*val*_, *X*_*test*_) = *Split*(*X*_*GADF*_, *ratio* = 1:1:2). 4. The parameters of DSCNN *θ* initialization. 5. Model training: For each epoch in the training phase: For each path *i* in DSC: (*α*_*ci*_,*α*_*hi*_,*α*_*wi*_) = *CCA*(*X*_*SCi_output*_) $X_{DSC\_output}=\sum_{i=1}^{3}\left(\alpha_{ci},\alpha_{hi},\alpha_{wi}\right)\cdot X_{SCi\_output}$ Calculate the output *logits* of DSCNN: *logits* = *DSCNN*(*X*_*train*_). Calculate the loss by using the logits and the *X*_*label*_: *loss* = *CrossentropyLoss*(*logits*, *X*_*label*_). Update the DSCNN parameters *θ*, through backpropagation. 6. Evaluate the model on the test set and calculate the predicted values: *Predict* = Trained_*DSCNN*(*X*_*test*_). 7. Return the predicted bearing health states *Y*_*predict*_.

## Experimental validation

### Dataset introduction

To verify the effectiveness of the proposed method, experiments use the Huazhong University of Science and Technology (HUST) [29] bearing dataset and the Harbin Institute of Technology (HIT) [30] aero-engine bearing dataset.

**Fig 6:**
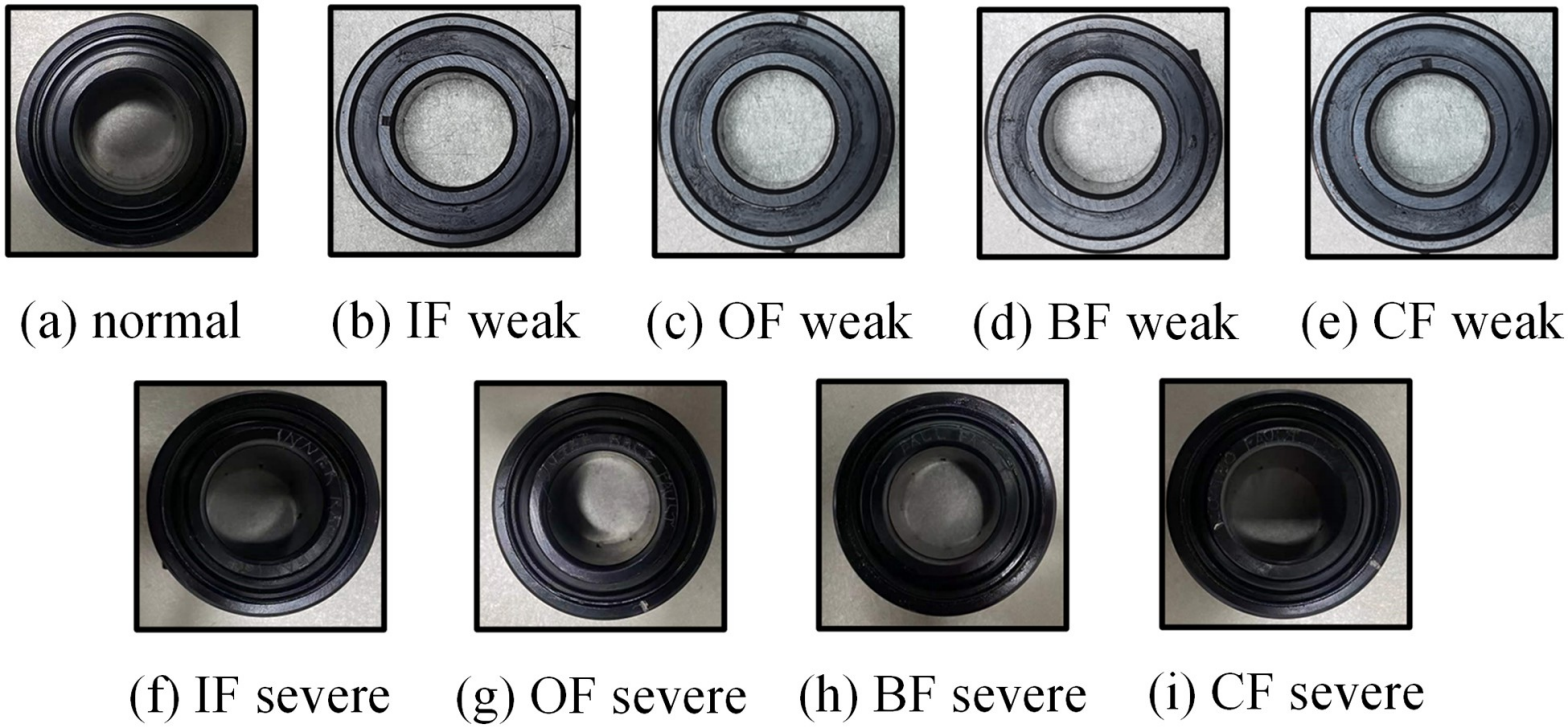
HUST bearing fault physical map.

The HUST bearing dataset includes normal states and various fault types, including inner race fault (IF), outer race fault (OF), ball fault (BF), and composite fault (CF). The bearing model is ER-16K, with a data sampling rate of 25.6 kHz. Vibration signals collected under different conditions (65Hz, 70Hz, 75Hz, 80Hz) are used for experimental validation. Fault types are shown in Fig 6.

**Fig 7:**
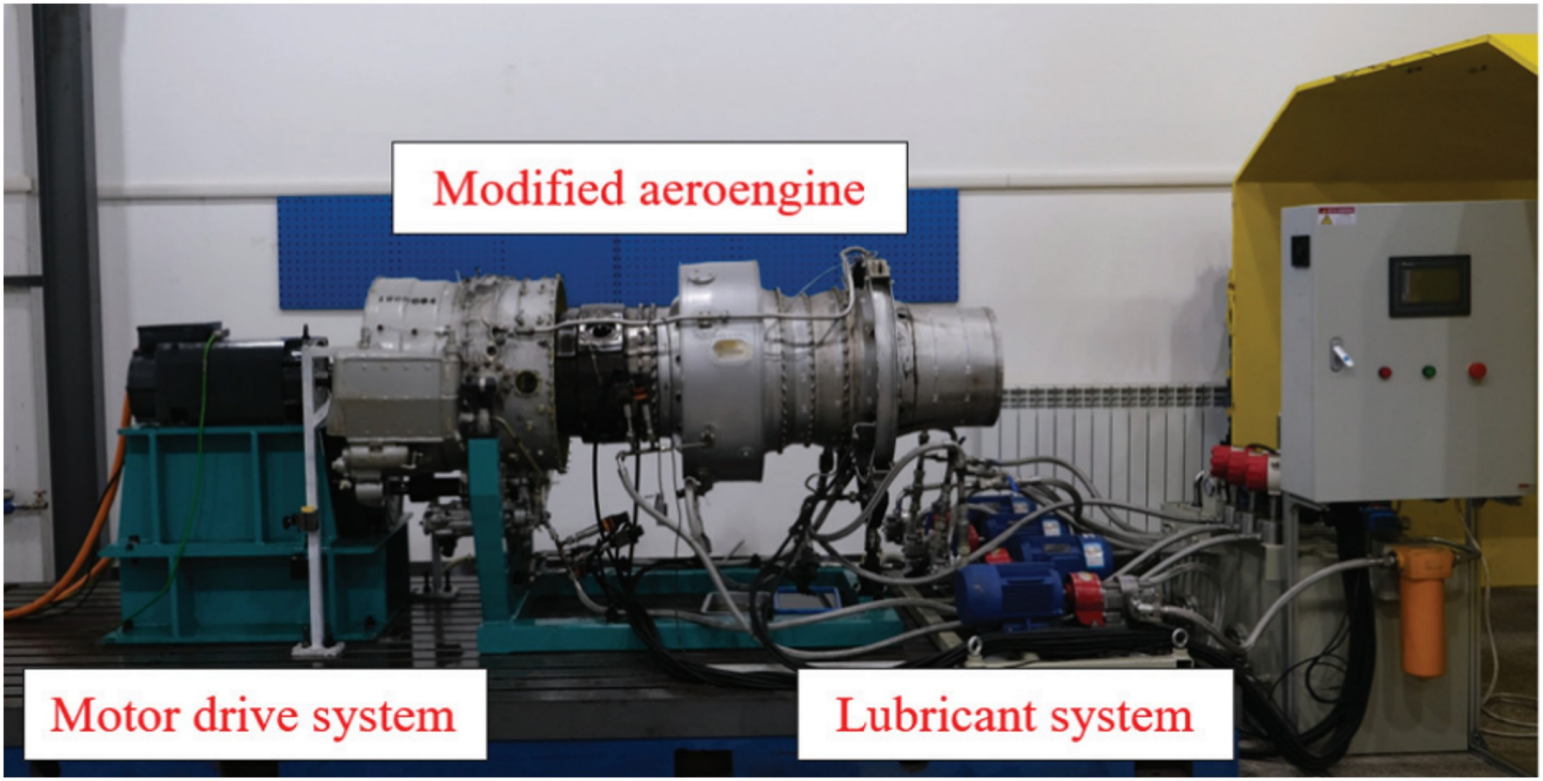
HIT aero-engine test rig.

**Fig 8:**
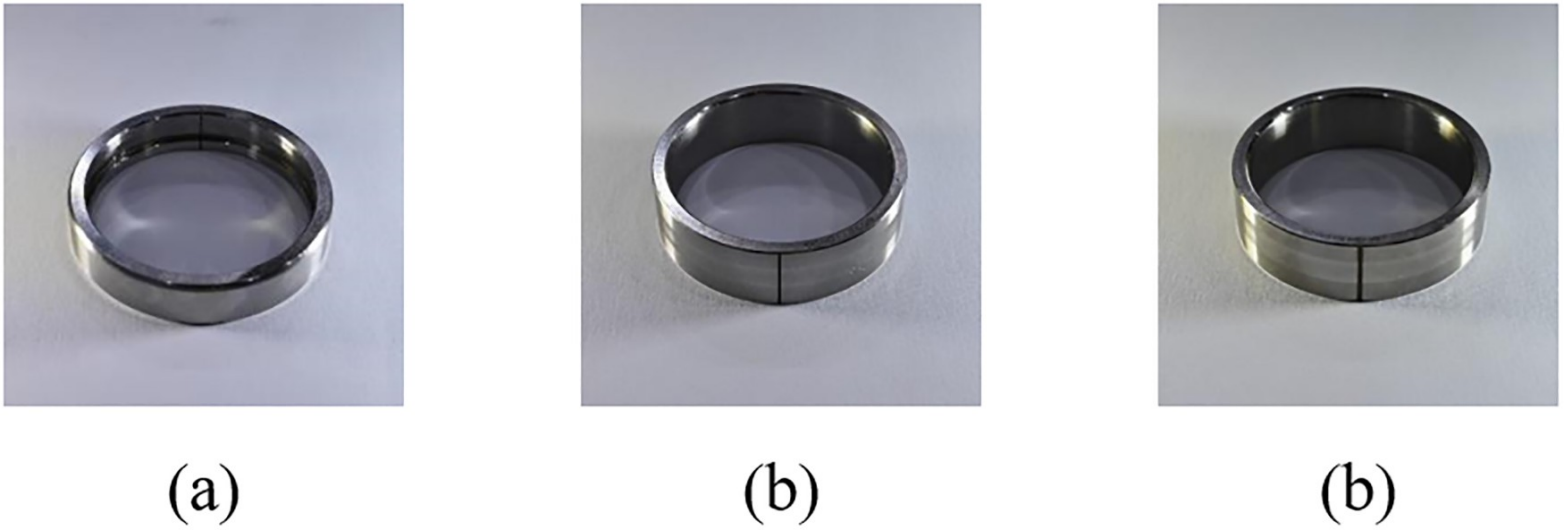
HIT bearing fault physical map.

The HIT aero-engine bearing test rig is a sophisticated testing system comprising an aero-engine, motor drive system, and lubrication oil system. The test rig structure is shown in Fig 7. The dataset includes healthy state, inner race fault, and outer race fault data, and bearing faults are shown in Fig 8. Sensors are strategically placed on the rotor and casing to accurately collect vibration displacement and acceleration signals at a sampling rate of 25 kHz. Dataset information is shown in Table 3. Experiments use data collected at 2000 r/min, 2500 r/min, and 3000 r/min. The HUST and HIT datasets were divided into train, validation, and test sets with sample sizes of 50, 50, and 100, respectively.

**Table 3:** Aero-engine bearing data.

Labels	Fault position	Depth of fault/mm	Length of fault/mm	Sample number
0	None	0	0	50/50/100
1	Inner race	0.5	0.5	50/50/100
2	Inner race	0.5	1.0	50/50/100
3	Outer race	0.5	0.5	50/50/100

Gaussian white noise [31] is a noise signal with statistical properties following a Gaussian distribution. In signal processing, Gaussian white noise refers to a random signal process where samples at any two equal interval time points are uncorrelated and follow a Gaussian distribution. To verify the noise resistance of the proposed method, Gaussian white noise with an intensity of -8 dB to 0 dB is added to the vibration signals. The formula can be represented as:

$${\text{SNR}}_{\text{dB}}=10{\text{log}}_{10}\left(\frac{P_{\text{signal}}}{P_{\text{noise}}}\right)$$

Where *P*_*signal*_ and *P*_noise_ are the original signal power and the noise power, respectively.

**Fig 9:**
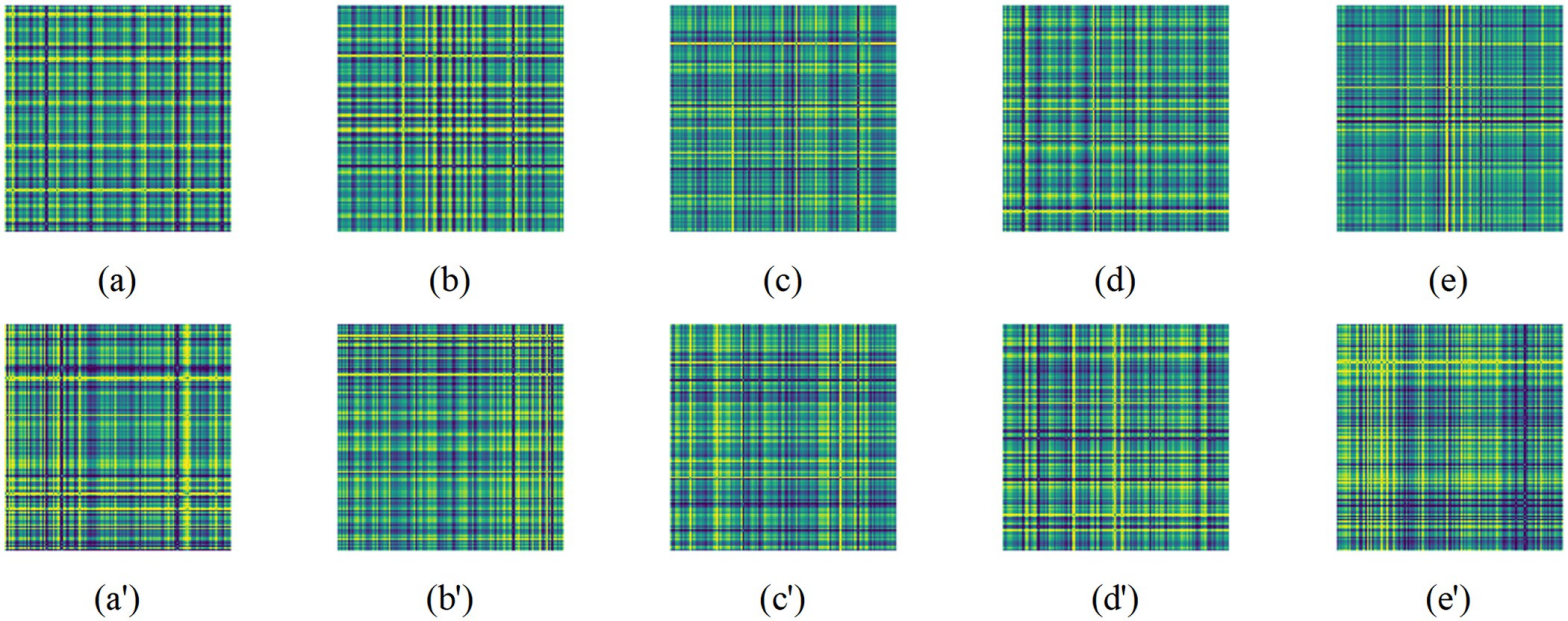
Comparison of GADF plots before and after adding noise. (a)-(e) denotes no noisy. (a’)-(e’) denotes adding noisy.

Fig 9 shows the comparison of HUST bearing data before and after adding noise. Without Gaussian white noise, the GADF images show clear and distinct signal features, making it easier for the model to identify the type of bearing fault. However, once noise is introduced, the originally clear signal patterns become blurred, replaced by a series of random and irregular interferences, significantly increasing the complexity of the signal.

### Experimental validation with HUST bearing data

To validate the proposed method, the DSCNN is compared with several advanced models—MSCNN [32], WMH-DRSSN [33], SC-MSCNN [34] and MAB-DrNet [35]—which have been recently effective in rolling bearing fault diagnosis. The HUST bearing dataset is used, with 50 training samples per category.

**Fig 10:**
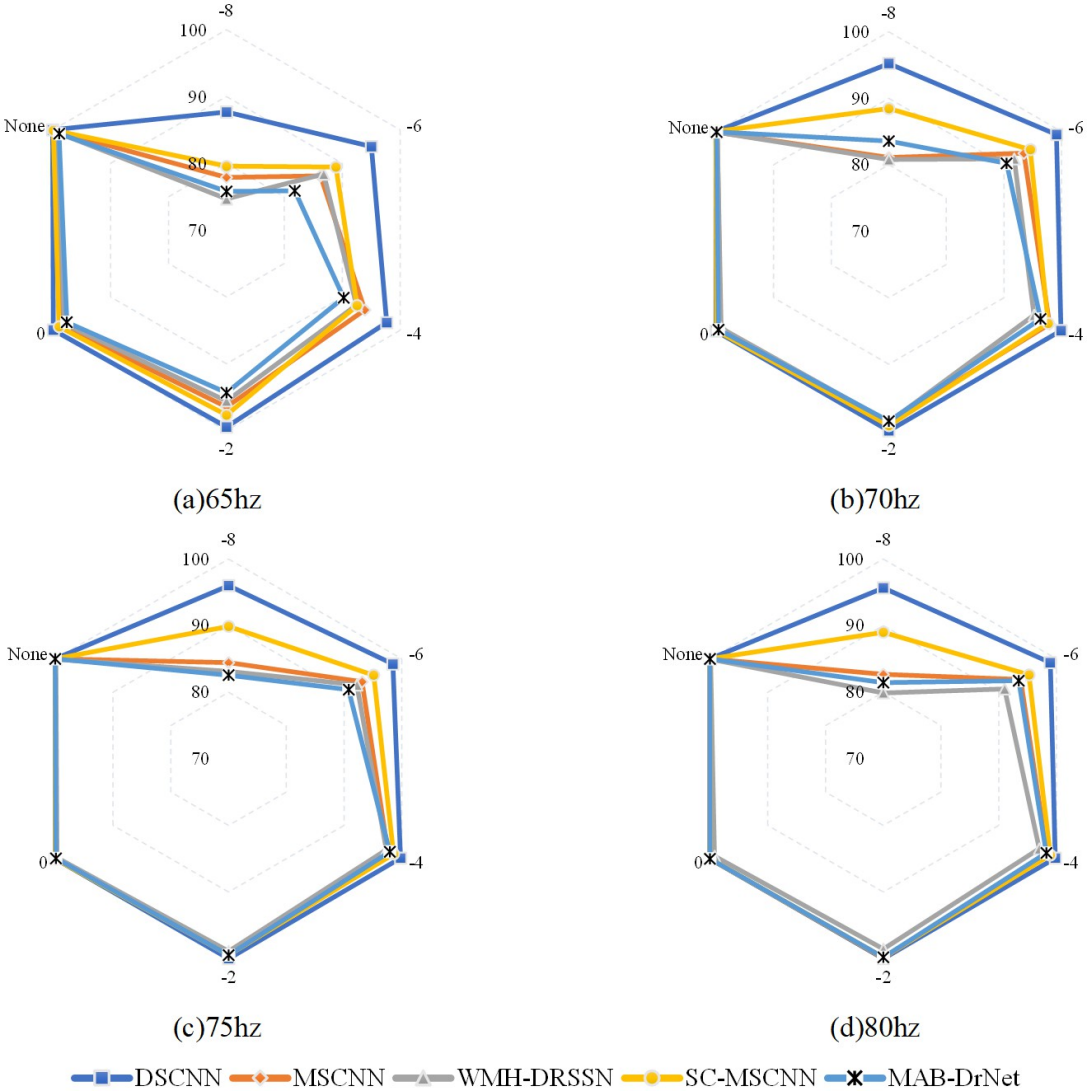
HUST classification results.

Fig 10 shows the performance of various models in rolling bearing fault diagnosis under different noise intensities. It is evident that DSCNN shows superior classification performance across all noise conditions, significantly outperforming other methods. The classification results highlight the robust noise resistance and stability of DSCNN in rolling bearing fault diagnosis. For example, with a noise intensity of -8 dB and using 70 Hz data, the classification accuracy of the DSCNN model is 95.22%, which is at least 6.7% higher than other models. As the noise intensity decreases, the classification accuracy of DSCNN increases further. As the noise intensity decreases to -2 dB, the classification accuracy of DSCNN reaches 100%, still maintaining its leading position in classification performance. This advantage is attributed to the unique network structure and efficient feature extraction strategy of DSCNN, which adopts the dynamic convolution technique to effectively filter the noise and capture the key information in the GADF image, thus achieving finer and more effective feature extraction.

Furthermore, DSCNN shows exceptional performance under other conditions, further confirming its superiority in rolling bearing fault diagnosis tasks. These results indicate that DSCNN not only excels under single conditions but also maintains stable performance across various scenarios, demonstrating its broad applicability and significant potential in practical applications.

**Fig 11:**
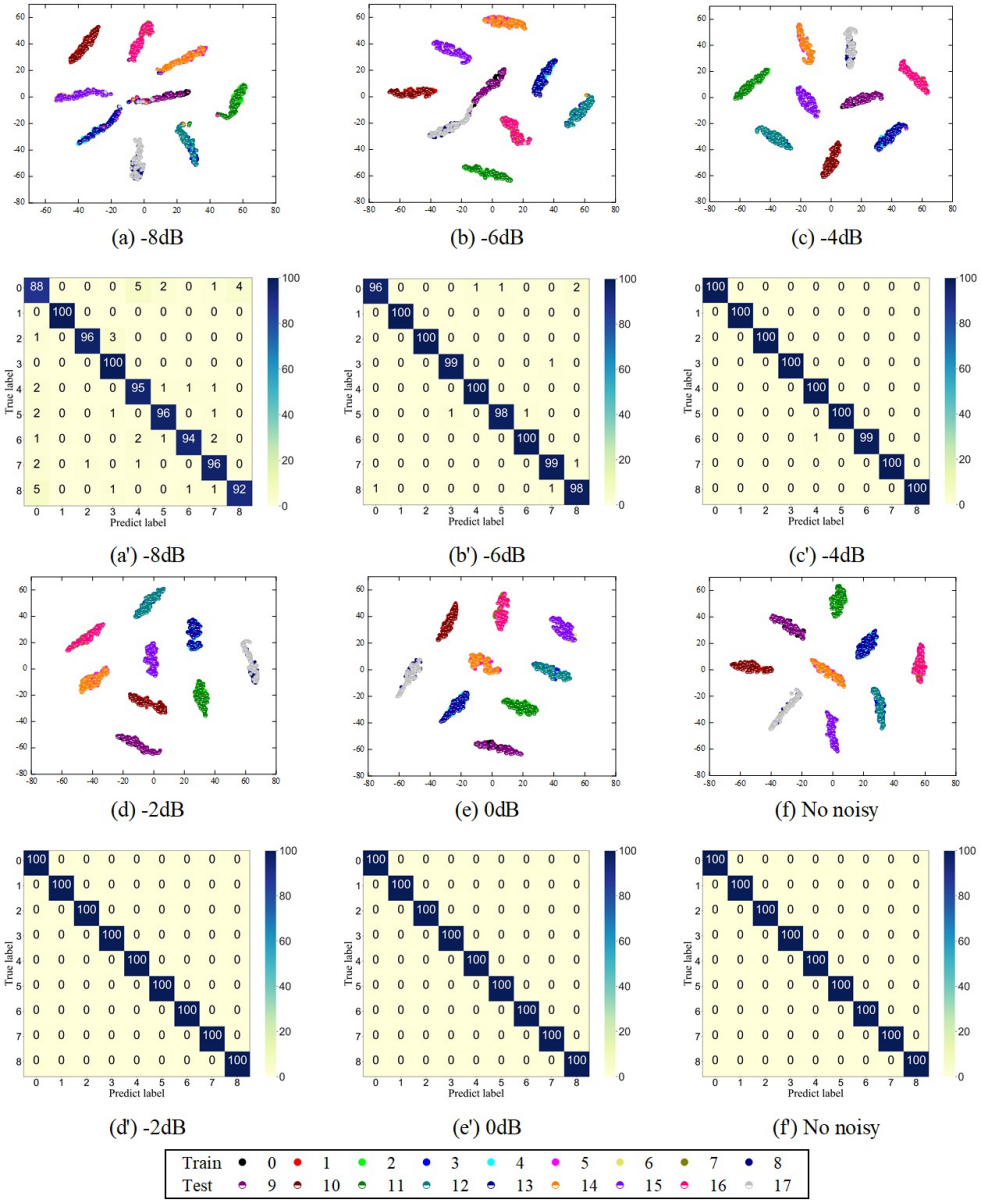
t-SNE visualization and confusion matrix.

Fig 11 shows the confusion matrix of the classification results of the DSCNN for different noise intensities and the t-distributed stochastic neighbor embedding (t-SNE) [36] visualization for the output features of the final layer. The confusion matrix provides an intuitive view of sample misclassification, while the t-SNE feature visualization reveals the distribution patterns of samples in the feature space. In the t-SNE visualization, each data point represents an independent sample, with colors and numerical labels indicating respective fault categories.

The visualization shows that under noise intensities below -4 dB, most samples are accurately classified. There is distinct separation between different categories in the feature space and tight clustering within the same category, indicating excellent overall clustering. The distribution pattern clearly shows that samples of the same category exhibit high similarity, while samples of different categories show significant differences. Although the overall clustering effect is satisfactory, the t-SNE visualization under high noise conditions reveals subtle overlapping phenomena, where boundaries between some categories are relatively blurred. This might be due to high similarity between these categories in the feature space, leading to less distinct differentiation. However, DSCNN still achieves the highest fault classification accuracy, demonstrating its strong performance and efficient feature extraction ability in classification tasks.

### Experimental validation with HIT bearing data

To further verify the ability of the proposed method to handle more complex and disturbing aero-engine bearing fault signals, the HIT bearing dataset is utilized for testing.

**Fig 12:**
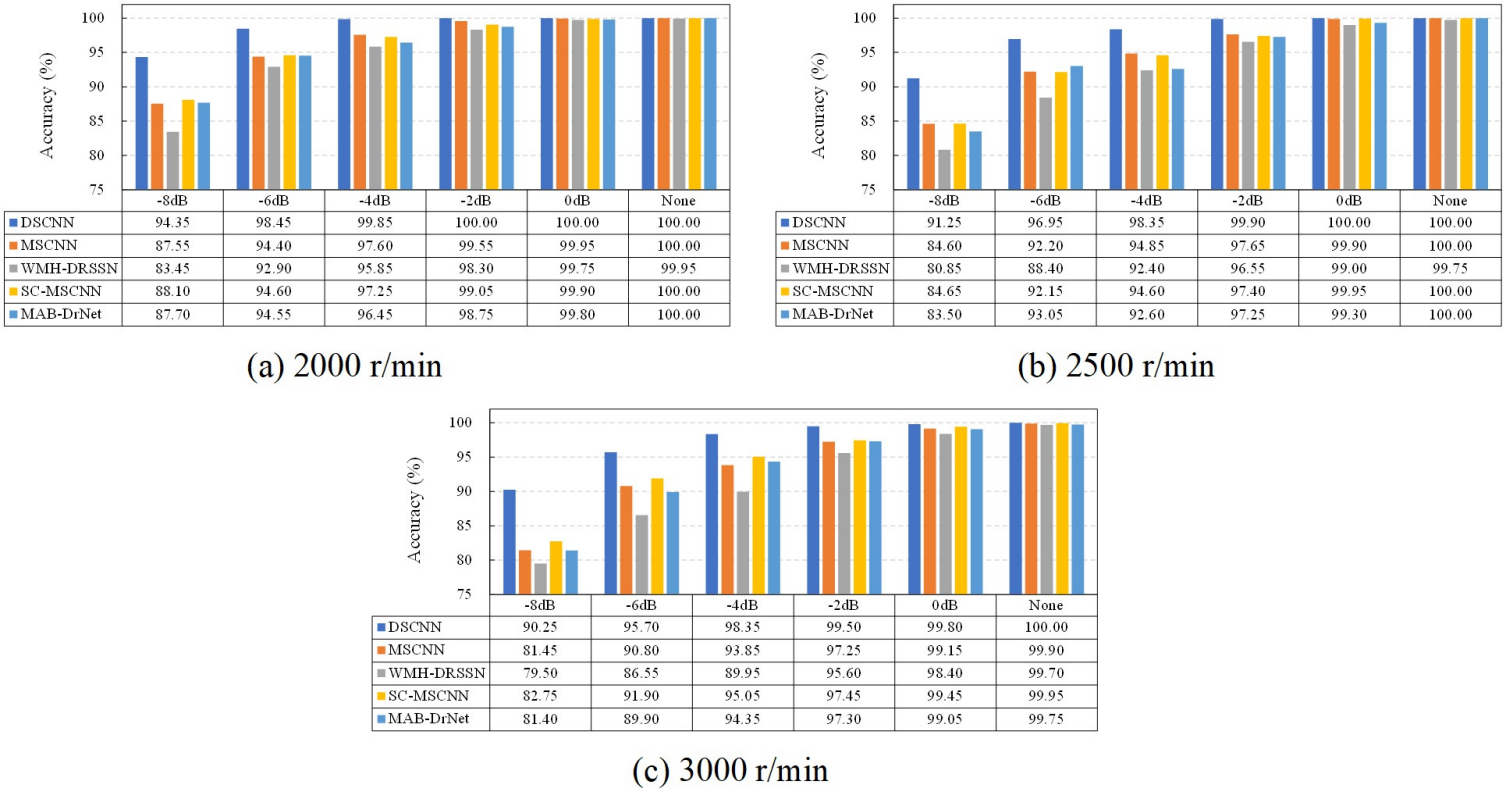
HIT classification results of different datasets.

Fig 12 shows the comparison of classification accuracy under different noise intensities. It is clear that as noise intensity decreases, the classification accuracy of all models improves. Particularly under low noise levels, the accuracy significantly increases, indicating a reduced impact of noise on classification results. DSCNN maintains high classification accuracy across all noise levels, despite fluctuations with speed changes. Taking the classification results of the bearing data at 3000 r/min as an example, it can be observed that the classification accuracy of the DSCNN model reaches 90.25% under the noise condition of -8 dB, which is 8.8% higher than the MSCNN, 10.75% higher than the WMH-DRSSN, 7.5% higher than the SC-MSCNN, and 8.85% higher than the MAB-DrNet. The classification accuracy of all models improved as the noise intensity decreased. Under the noise condition of 0 dB, the classification accuracy of DSCNN is more than 0.35% higher than the other models, which still maintains its leading position, although the advantage is narrowed.

Overall, DSCNN performs better than other comparative methods, highlighting its strong recognition ability for complex fault signals and robustness under high interference conditions. Even under high noise levels, DSCNN maintains high accuracy, proving its strong noise resistance. This result not only reflects the theoretical superiority of DSCNN, but also verifies its reliability in practical applications.

**Fig 13:**
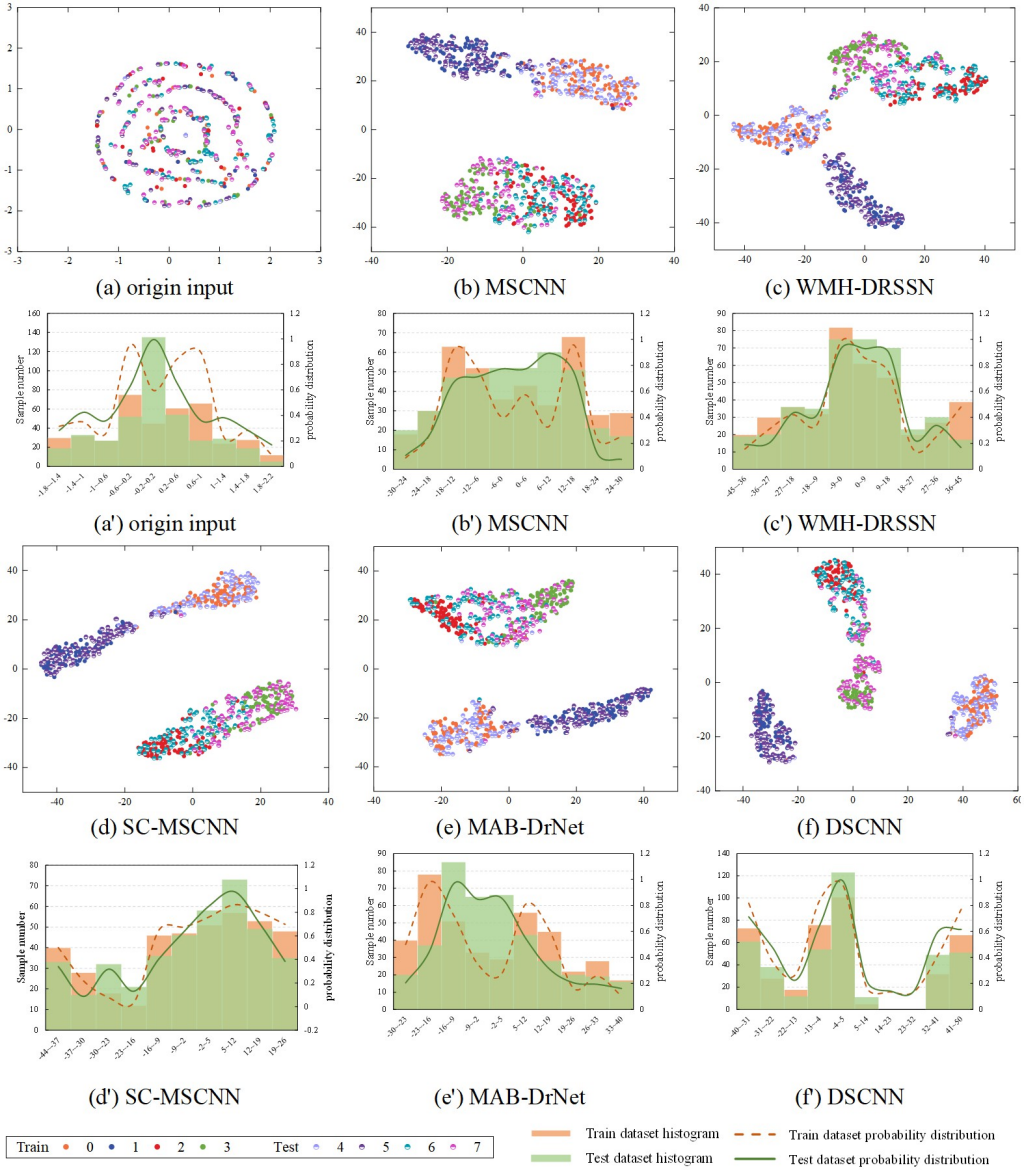
Data visualization results on the HIT dataset. (a)-(f) represent the t-SNE visualization result. (a’)-(f’) represent probability density estimation curves and histograms for different models.

To visually verify the classification effect of DSCNN, t-SNE visualization is used to reveal the distribution of output data in the feature space across different models, as shown in Fig 13. Comparing the t-SNE plots of the output data at the final network layer between DSCNN and other methods, it is clear that DSCNN excels in feature separability. The t-SNE plots of DSCNN show clearer category boundaries compared to the t-SNE plots of the other methods. In the plots of other methods, category boundaries are blurred, making precise classification challenging. In contrast, the t-SNE plot of DSCNN shows clear category boundaries, with higher matching between training and testing data in the feature space, indicating the model has high efficiency in recognizing and distinguishing different fault types.

When analyzing the kernel probability density distribution presented in Fig 13, other comparative methods show significant errors in estimating the feature space differences between training and testing data, leading to poor domain matching. This result clearly points out that performing fault diagnosis tasks under high noise environments is highly challenging. These methods often fail to accurately capture the essential differences between source and target domains under strong noise interference, thus affecting the diagnostic accuracy of the model.

## 5. Conclusion

To address the issue of inadequate feature extraction for fault characteristics under small sample sizes and strong noise conditions, a rolling bearing fault diagnosis method based on GADF and DSC modules is proposed. The GADF and DSC modules effectively capture the nonlinear relationships in bearing vibration signals and dynamically adjust convolutional kernel weights to adapt to different data distributions, enhancing model generalization and diagnostic accuracy. Additionally, the proposed cooperative compensation attention mechanism simultaneously considers the correlation of features in channel and spatial dimensions, comprehensively evaluates the importance of each feature, and dynamically adjusts DSC module weights, achieving feature complementarity and optimization. This mechanism further enhances the selectivity and sensitivity of the model to key features and improves noise immunity.

Experimental validation on HUST and HIT bearing datasets shows that DSCNN can more accurately identify and classify different types of bearing faults compared to other methods, with stronger generalization and robustness. Even under high noise conditions, DSCNN maintains excellent diagnostic performance with classification accuracies of more than 90% under different datasets for the majority of the datasets, which proves its robustness.

In summary, the proposed method effectively enhances the feature extraction ability for rolling bearing fault diagnosis under small sample and high noise conditions, providing new ideas and methods for rolling bearing fault diagnosis.
